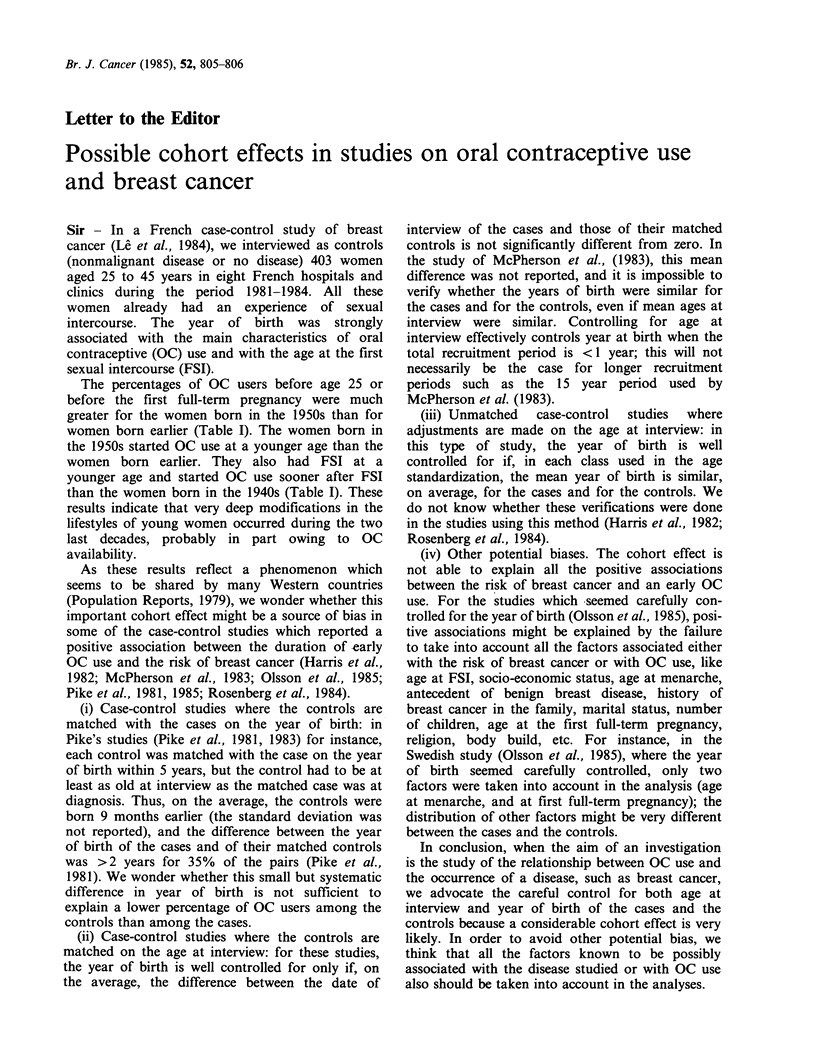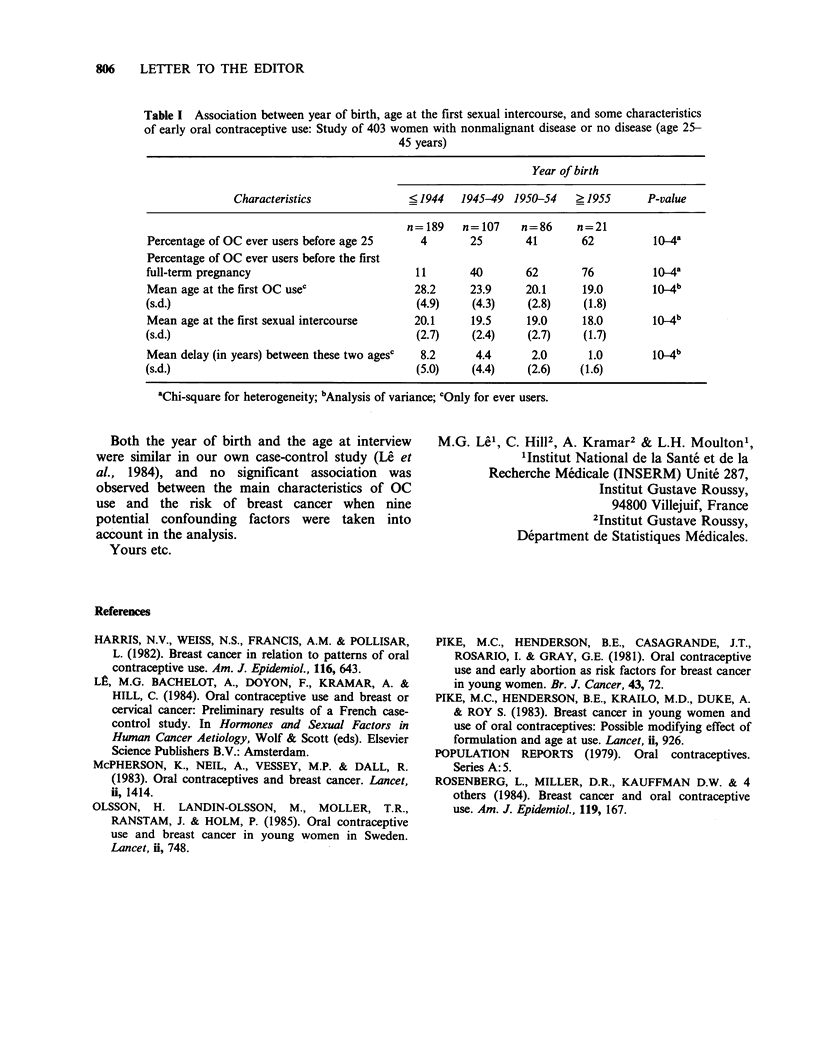# Possible cohort effects in studies on oral contraceptive use and breast cancer.

**DOI:** 10.1038/bjc.1985.263

**Published:** 1985-11

**Authors:** M. G. Lê, C. Hill, A. Kramar, L. H. Moulton


*Br. J. Cancer* (1985), **52**, 805–806

### Letter to the Editor

### Possible cohort effects in studies on oral contraceptive use and breast cancer

**Sir** – In a French case-control study of breast cancer (Lê *et al.*, 1984), we interviewed as controls (nonmalignant disease or no disease) 403 women aged 25 to 45 years in eight French hospitals and clinics during the period 1981–1984. All these women already had an experience of sexual intercourse. The year of birth was strongly associated with the main characteristics of oral contraceptive (OC) use and with the age at the first sexual intercourse (FSI).

The percentages of OC users before age 25 or before the first full-term pregnancy were much greater for the women born in the 1950s than for women born earlier (Table I). The women born in the 1950s started OC use at a younger age than the women born earlier. They also had FSI at a younger age and started OC use sooner after FSI than the women born in the 1940s (Table I). These results indicate that very deep modifications in the lifestyles of young women occurred during the two last decades, probably in part owing to OC availability.

As these results reflect a phenomenon which seems to be shared by many Western countries (Population Reports, 1979), we wonder whether this important cohort effect might be a source of bias in some of the case-control studies which reported a positive association between the duration of early OC use and the risk of breast cancer (Harris *et al.*, 1982; McPherson *et al.*, 1983; Olsson *et al.*, 1985; Pike *et al.*, 1981, 1985; Rosenberg *et al.*, 1984).

(i) Case-control studies where the controls are matched with the cases on the year of birth: in Pike's studies (Pike *et al.*, 1981, 1983) for instance, each control was matched with the case on the year of birth within 5 years, but the control had to be at least as old at interview as the matched case was at diagnosis. Thus, on the average, the controls were born 9 months earlier (the standard deviation was not reported), and the difference between the year of birth of the cases and of their matched controls was >2 years for 35% of the pairs (Pike *et al.*, 1981). We wonder whether this small but systematic difference in year of birth is not sufficient to explain a lower percentage of OC users among the controls than among the cases.

(ii) Case-control studies where the controls are matched on the age at interview: for these studies, the year of birth is well controlled for only if, on the average, the difference between the date of interview of the cases and those of their matched controls is not significantly different from zero. In the study of McPherson *et al.*, (1983), this mean difference was not reported, and it is impossible to verify whether the years of birth were similar for the cases and for the controls, even if mean ages at interview were similar. Controlling for age at interview effectively controls year at birth when the total recruitment period is <1 year; this will not necessarily be the case for longer recruitment periods such as the 15 year period used by McPherson *et al.* (1983).

(iii) Unmatched case-control studies where adjustments are made on the age at interview: in this type of study, the year of birth is well controlled for if, in each class used in the age standardization, the mean year of birth is similar, on average, for the cases and for the controls. We do not know whether these verifications were done in the studies using this method (Harris *et al.*, 1982; Rosenberg *et al.*, 1984).

(iv) Other potential biases. The cohort effect is not able to explain all the positive associations between the risk of breast cancer and an early OC use. For the studies which seemed carefully controlled for the year of birth (Olsson *et al.*, 1985), positive associations might be explained by the failure to take into account all the factors associated either with the risk of breast cancer or with OC use, like age at FSI, socio-economic status, age at menarche, antecedent of benign breast disease, history of breast cancer in the family, marital status, number of children, age at the first full-term pregnancy, religion, body build, etc. For instance, in the Swedish study (Olsson *et al.*, 1985), where the year of birth seemed carefully controlled, only two factors were taken into account in the analysis (age at menarche, and at first full-term pregnancy); the distribution of other factors might be very different between the cases and the controls.

In conclusion, when the aim of an investigation is the study of the relationship between OC use and the occurrence of a disease, such as breast cancer, we advocate the careful control for both age at interview and year of birth of the cases and the controls because a considerable cohort effect is very likely. In order to avoid other potential bias, we think that all the factors known to be possibly associated with the disease studied or with OC use also should be taken into account in the analyses.

**Table I** Association between year of birth, age at the first sexual intercourse, and some characteristics of early oral contraceptive use: Study of 403 women with nonmalignant disease or no disease (age 25–45 years)

| *Characteristics* | *Year of birth* ≦1944 | 1945–49 | 1950–54 | ≧1955 | *P-value* |
|---|---|---|---|---|---|
| | $n=189$ | $n=107$ | $n=86$ | $n=21$ | |
| Percentage of OC ever users before age 25 | 4 | 25 | 41 | 62 | 10–4[a] |
| Percentage of OC ever users before the first full-term pregnancy | 11 | 40 | 62 | 76 | 10–4[a] |
| Mean age at the first OC use[c] | 28.2 | 23.9 | 20.1 | 19.0 | 10–4[b] |
| (s.d.) | (4.9) | (4.3) | (2.8) | (1.8) | |
| Mean age at the first sexual intercourse | 20.1 | 19.5 | 19.0 | 18.0 | 10–4[b] |
| (s.d.) | (2.7) | (2.4) | (2.7) | (1.7) | |
| Mean delay (in years) between these two ages[c] | 8.2 | 4.4 | 2.0 | 1.0 | 10–4[b] |
| (s.d.) | (5.0) | (4.4) | (2.6) | (1.6) | |

[a]Chi-square for heterogeneity; [b]Analysis of variance; [c]Only for ever users.

Both the year of birth and the age at interview were similar in our own case-control study (Lê *et al.*, 1984), and no significant association was observed between the main characteristics of OC use and the risk of breast cancer when nine potential confounding factors were taken into account in the analysis.

Yours etc.

M.G. Lê[1], C. Hill[2], A. Kramar[2] & L.H. Moulton[1],
[1]Institut National de la Santé et de la Recherche Médicale (INSERM) Unité 287,
Institut Gustave Roussy,
94800 Villejuif, France
[2]Institut Gustave Roussy,
Départment de Statistiques Médicales.

### References

HARRIS, N.V., WEISS, N.S., FRANCIS, A.M. & POLLISAR, L. (1982). Breast cancer in relation to patterns of oral contraceptive use. *Am. J. Epidemiol.*, **116,** 643.

LÊ, M.G. BACHELOT, A., DOYON, F., KRAMAR, A. & HILL, C. (1984). Oral contraceptive use and breast or cervical cancer: Preliminary results of a French case-control study. In *Hormones and Sexual Factors in Human Cancer Aetiology,* Wolf & Scott (eds). Elsevier Science Publishers B.V.: Amsterdam.

McPHERSON, K., NEIL, A., VESSEY, M.P. & DALL, R. (1983). Oral contraceptives and breast cancer. *Lancet,* **ii,** 1414.

OLSSON, H. LANDIN-OLSSON, M., MOLLER, T.R., RANSTAM, J. & HOLM, P. (1985). Oral contraceptive use and breast cancer in young women in Sweden. *Lancet,* **ii,** 748.

PIKE, M.C., HENDERSON, B.E., CASAGRANDE, J.T., ROSARIO, I. & GRAY, G.E. (1981). Oral contraceptive use and early abortion as risk factors for breast cancer in young women. *Br. J. Cancer,* **43,** 72.

PIKE, M.C., HENDERSON, B.E., KRAILO, M.D., DUKE, A. & ROY S. (1983). Breast cancer in young women and use of oral contraceptives: Possible modifying effect of formulation and age at use. *Lancet,* **ii,** 926.

POPULATION REPORTS (1979). Oral contraceptives. Series A:5.

ROSENBERG, L., MILLER, D.R., KAUFFMAN D.W. & 4 others (1984). Breast cancer and oral contraceptive use. *Am. J. Epidemiol.,* **119,** 167.